# Prevalence of household food insecurity among a healthy Iranian population: A systematic review and meta-analysis

**DOI:** 10.3389/fnut.2022.1006543

**Published:** 2022-11-15

**Authors:** Pishva Arzhang, S. Haniye Abbasi, Peyman Sarsangi, Mahsa Malekahmadi, Mahlagha Nikbaf-Shandiz, Nick Bellissimo, Leila Azadbakht

**Affiliations:** ^1^Qods Hospital, Kermanshah University of Medical Sciences, Kermanshah, Iran; ^2^Department of Community Nutrition, School of Nutritional Sciences and Dietetics, Tehran University of Medical Sciences, Tehran, Iran; ^3^Imam Khomeini Hospital Complex, Tehran University of Medical Sciences, Tehran, Iran; ^4^Student Research Committee, Tabriz University of Medical Sciences, Tabriz, Iran; ^5^School of Nutrition, Toronto Metropolitan University, Toronto, ON, Canada; ^6^Diabetes Research Center, Endocrinology and Metabolism Clinical Sciences Institute, Tehran University of Medical Sciences, Tehran, Iran

**Keywords:** food insecurity, food security, Iran, prevalence, food supply

## Abstract

**Background:**

Food security is a fundamental human right that must be upheld to preserve excellent general welfare, and mental, physical, and social health. However, according to the United Nations Food and Agriculture Organization (FAO) report in 2020, the level of food insecurity in the world is increasing.

**Objective:**

Determining the prevalence of food insecurity in Iran will be beneficial for Iran and other low-middle-income countries.

**Methods:**

We searched both English and Persian (Iranian) databases including PubMed, Scopus, Web of Science, Google Scholar, SID, Irandoc, Magiran, Civilica, and Iranian Medical Sciences Theses System from 01 January 1990 to 01 February 2022. Observational studies that reported the prevalence of household food insecurity among a healthy Iranian population and assessed food insecurity at the individual or household level using validated questionnaires were included.

**Results:**

One hundred six studies and/or theses with a total of 152, 300 participants met the review criteria. Our analyses demonstrated that the prevalence of food insecurity among the healthy Iranian population was 55.9% (95% CI: 52.6–59.2%) and the highest prevalence of food insecurity was in the western regions with 64.8% (95% CI: 57.7–72.0%). Subgroup analyses showed that food insecurity among women at 51.3% (95% CI: 45.1–57.6%) and rural inhabitants at 66.1% (95% CI: 58.8–73.4%) was significantly higher than men at 47.8% (95% CI: 41.8–53.8%) and urban residents at 47.1% (95% CI: 44.1–50.0%), respectively. Among the age groups, the highest prevalence of food insecurity was in adults at 56.5% (95% CI: 51.7–61.2%).

**Conclusion:**

The prevalence of food insecurity in a healthy Iranian population was higher than the global average. Women, rural residents, and residents of the western regions of Iran had a higher prevalence of food insecurity. These groups should be prioritized in programs to reduce the prevalence of food insecurity in Iran.

**Systematic review registration:**

www.crd.york.ac.uk/PROSPERO, identifier: CRD42022328473.

## Introduction

Household food security has been defined as all members having adequate access to safe food through socially acceptable means for active and healthy life (1). Limited or uncertain sources of adequate and safe nutritional foods or the limited or uncertain ability to acquire food lead to food insecurity (FI). FI is a public health challenge around the world and millions of households are struggling to maintain or achieve food security, which includes availability, access, utilization, and ability (2, 3). It is not considered a static state and is on a spectrum (3). According to the United Nations in 2021, there is a higher prevalence of FI since the beginning of the COVID-19 pandemic compared to before the pandemic (4). The prevalence of household FI in north Iran has been estimated to be ~50.8% in total with 43.2% mild FI, 6.5% moderate FI, and 1.1% severe FI (5), which may have increased in recent years due to the COVID-19 pandemic and other economic and social issues.

Various methods are used to evaluate FI, two of which are used the most: (1) estimation of food consumption and (2) estimation of the cost of food preparation (6). These methods have limitations due to the cost, participant burden, and the need for an expert interviewer. Recently, “experiential” or “perception-based” methods have been used to measure FI (7). Under these methods, individual experience of FI (i.e., access) is measured through a survey and summarized in a scale. The Household Food Security Survey Module (HFSSM) has been consistently validated as a reliable and significant perception-based measure of FI in the United States (8). In Iran, FI is mainly investigated by measuring the adequacy of energy and nutrients using food questionnaires or by estimating the poverty line from income and expenditure surveys (6). FI is a threat to the health and survival of individuals within communities and can have both short- and long-term effects (9). Several factors influence the prevalence of FI including population growth, industrialization of communities, growing migration from rural to urban areas, inadequate levels of education, wars and economic sanctions by governments, pandemics or endemic diseases, and weather changes (10–12). In addition, FI can include a wide range of health-related conditions from undernutrition to overnutrition. Undernutrition includes wasting, underweight, stunting, anemia, and other diseases associated with nutritional deficiencies. Overnutrition can lead to chronic conditions such as obesity, diabetes, fatty liver, hypertension, poor mental health, and metabolic syndrome (13–17). As a result, a large burden is faced by the economy and society (5).

Several studies have been conducted to investigate the prevalence, causes, and types of FI around the world and in Iran. According to a meta-analysis from West Africa, the prevalence of FI in the rural population was ~60.91% and the highest prevalence was among large families, female-headed households, and low-income households with low education levels (10). Several systematic reviews and meta-analyses have been conducted in Iran (6, 11, 12). In a meta-analysis from 2017, the prevalence of FI was 49.2% based on studies that evaluated FI using questionnaires (12). Since the publication of the previous meta-analysis, many studies have been published evaluating the status of FI in Iran. Considering this, a new meta-analysis is warranted to provide updated results and summarize recent findings. Therefore, the objective of this systematic review and meta-analysis is to update the findings and conduct a more comprehensive review of the prevalence of FI with the consideration of multiple subgroups and other factors.

## Method

This systematic review and meta-analysis were conducted based on the Preferred Reporting Items for Systematic Review and Meta-Analysis statement (PRISMA) guideline (18). The protocol of the present study was registered on the PROSPERO website (www.crd.york.ac.uk/PROSPERO) (PROSPERO registration number = CRD42022328473).

### Search strategy and information sources

International scientific databases including Web of Science, PubMed, Scopus, Google Scholar, and Iranian databases (HYPERLINKs: “https://www.sid.ir/fa/journal/,” “https://ganj.irandoc.ac.ir/,” “https://www.magiran.com/paperadvancedsearch,” “http://thesis.research.ac.ir/faces/home.jspx,” and “https://civilica.com/”) were searched from 01 January 1990 to 01 February 2022 to identify relevant studies. The detailed search strategy is shown in Supplementary Table 1.

### Inclusion and exclusion criteria

Inclusion criteria were (a) observational studies (cross-sectional and cohort studies), (b) studies published between 1990 and 2022, (c) studies with a healthy Iranian population, (d) studies that reported the prevalence and/or at least one associated factor with household FI, and (e) studies that assessed FI at the individual or household level using validated questionnaires. Gray literature from credible sources was included. Exclusion criteria were intervention studies, abstract studies, studies performed on pregnant women, and populations with evidence of specific diseases.

### Quality assessment

The Joanna Briggs Institute (JBI) quality assessment checklist was used to perform the quality assessment of the published articles (19). Two independent researchers (HA and PS) conducted a quality assessment for each of the included studies. Studies that received scores of ≥5 were categorized as low risk or good quality and studies with scores of ≤4 were categorized as high risk or poor quality.

### Study selection and data extraction

Two independent reviewers (HA and PS) screened the studies based on the eligibility criteria. The abstracts and titles of the studies were reviewed, and the included full-text studies were evaluated. Any disagreements were addressed by a third reviewer's decision.

The following data were extracted from the included studies: the name of the first author, publication year, study design, province, geographical zone, urban/rural, gender, sample size, and prevalence of household food insecurity and relevant FI factors.

### Data synthesis and statistical analysis

Pooled estimates for the magnitude of household food insecurity were calculated using a random effects model and metaprop module in Stata 14.2. The reason for using a random effects model was to attempt to account for the methodological differences within the included articles. A binomial distribution formula was used to estimate standard errors for each sample (20). The Egger's test and Trim and Fill test were used to evaluate publication bias. A funnel plot of symmetry visualized publication bias graphically. Evidence of statistically significant publication bias was considered a *p-*value < 0.05. Sensitivity analyses were performed by removing the studies that showed evidence of potential publication bias (21). The heterogeneity between studies was assessed using the I^2^ test (22). Subgroup analyses were conducted based on geographic region, age groups, type of questionnaire, quality of studies, location, and gender. Using meta-regression analysis, we examined the presence of any linear relationship between observed effect size and time. Evidence of a significant difference within the subgroups by the test of group difference was considered *P* < 0.05.

## Results

**Figure 1:**
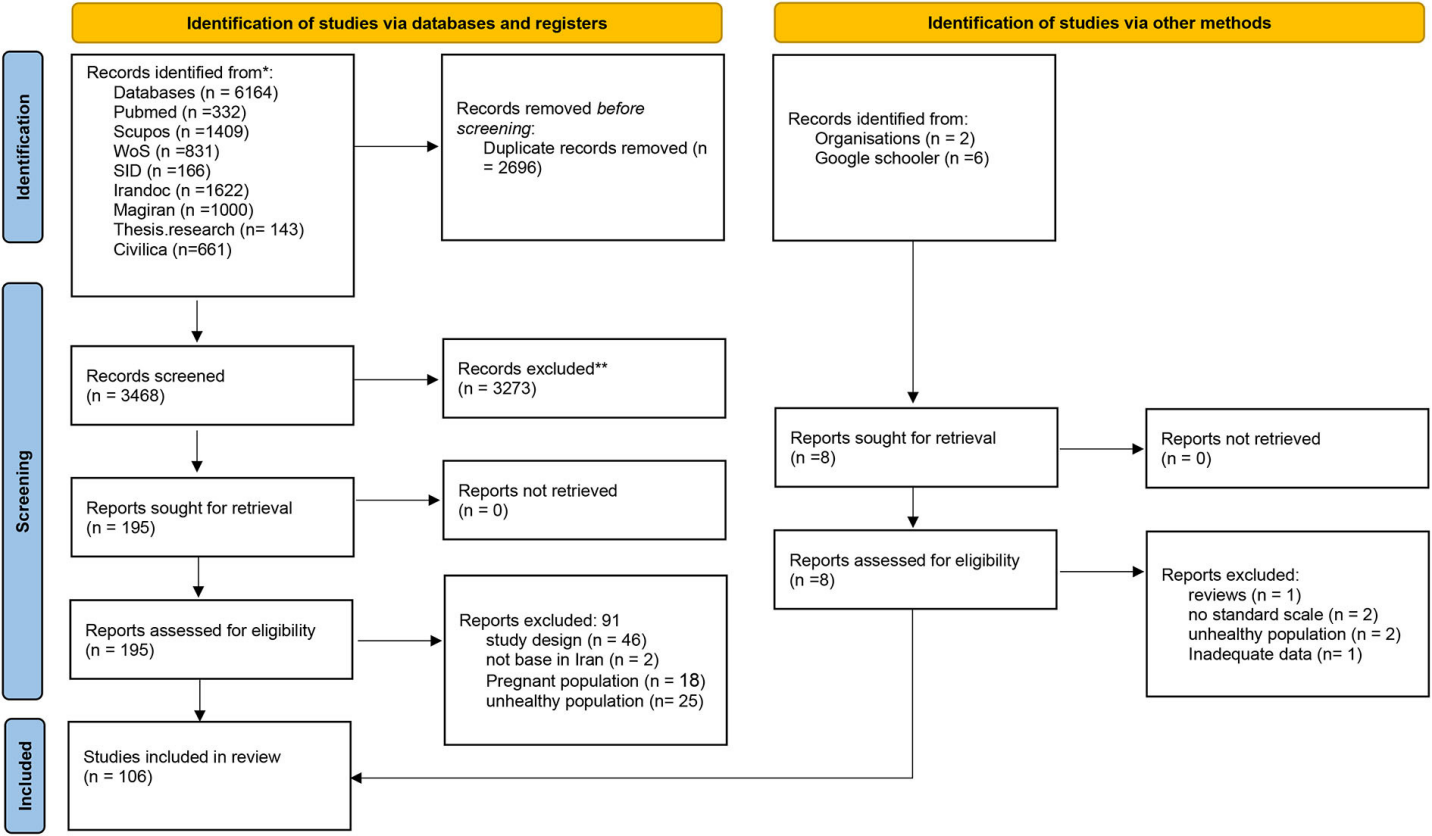
PRISMA flow diagram for study selection from: Page et al. (23). For more information, visit: http://www.prisma-statement.org/.

A total of 6,164 articles and theses were retrieved from the initial database search. After the removal of duplicates, 3,468 studies remained to be screened. Title and abstracts were screened yielding 195 studies for full-text review. One hundred six studies (96 cross-sectional articles and 10 theses) published between 2010 and 2021 including a total of 152,300 participants were included after a full-text review and for data extraction. Study selection is presented in the PRISMA flowchart (Figure 1).

The characteristics of the included studies are shown in Table 1. Since some of the studies reported FI in different locations or genders, we considered them as separate studies. Of the included studies, 51 examined FI in urban areas, 41 in rural areas, and 14 in both areas. Included studies also investigated FI among men and women (*n* = 70), men (n= 20), and women (n= 35). In addition, studies were conducted on different age groups, including children (*n* = 14), adolescents (*n* = 6), adults (*n* = 45), and older adults (*n* = 9). Studies were conducted in several different locations including the central (*n* = 36), western (*n* = 34), southern (*n* = 16), northern (*n* = 7), and eastern (*n* = 11) parts of the country. Different questionnaires were used to assess FI including the United States Department of Agriculture 18-item Household Food Security Survey Module (USDA 18-item questionnaire) (*n* = 48), Household Food Insecurity Access Scale (HFIAS) (*n* = 32), US Department of Agriculture 6-item Household Food Security Survey Module (USDA 6-item) (*n* = 12), Radimer-Cornell (*n* = 6), Farsi Current Population Survey-Food Security Survey Module (FaCPS-FSSM) (*n* = 3), and the Coping Strategies Index (*n* = 3).

**Table 1:** Characteristics of the included studies in the systematic review and meta-analysis.

**References**	**Year of publication**	**Study design**	**Province**	**Geographic zone**	**Urban/rural**	**Gender**	**Sample size**
Mohammadzadeh et al. (24)	2010	Cross-sectional	Isfahan	Central Iran	Urban	Both	580
Asgharian Dastnaei et al. (25)	2013	Cross-sectional	Chaharmahal and Bakhtiari	Central Iran	Rural	Both	343
Afshar et al. (26)	2018	Cross-sectional	Alborz	Central Iran	Urban	Both	677
Jozi et al. (27)	2020	Cross-sectional	Zanjan	Western Iran	Rural	Both	353
Narmaki et al. (28)	2016	Cross-sectional	Tehran	Central Iran	Urban	Female	397
Motlagh et al. (29)	2019	Cross-sectional	Tehran	Central Iran	Urban	Female	452
Mokari-Yamchi et al. (30)	2020	Cross-sectional	East Azarbaijan	Western Iran	Rural	Both	404
Rezazadeh et al. (31)	2016	Cross-sectional	West Azerbaijan	Western Iran	Urban	Both	723
Ahmadihoseini et al. (32)	2019	Cross-sectional	Khorasan razavi	Eastern Iran	Urban	Both	240
Khosravipour et al. (33)	2017	Cross-sectional	Khuzestan	Western Iran	both	Both	198
Alipour et al. (34)	2016	Cross-sectional	East Azarbaijan	Western Iran	Urban	Both	330
Daneshzad et al. (35)	2021	Cross-sectional	Tehran	Central Iran	Urban	Female	788
Parvin et al. (36)	2020	Cross-sectional	Kohgiluyeh and Boyer-Ahmad	Southern Iran	Urban	Female	400
Tabrizi et al. (37)	2018	Cross-sectional	East Azarbaijan	Western Iran	Urban	Both	1,386
Rezaee et al. (38)	2021	Cross-sectional	Golestan	Northern Iran	Rural	Both	291
Saadi et al. (39)	2014	Cross-sectional	Kurdistan	Western Iran	Rural	Both	200
Saadi et al. (40)	2014	Cross-sectional	Hamedan	Western Iran	Rural	Female	92
Gholizadeh et al. (41)	2017	Cross-sectional	Kermanshah	Western Iran	Rural	Both	258
Farzaneh et al. (42)	2017	Cross-sectional	East Azarbaijan	Western Iran	Urban	Both	480
Fami et al. (43)	2021	Cross-sectional	Tehran	Central Iran	Urban	Female	750
Jamini et al. (44)	2017	Cross-sectional	Kermanshah	Western Iran	Rural	Both	180
Jamini et al. (45)	2017	Cross-sectional	Kermanshah	Western Iran	Rural	Both	175
Sharafkhani et al. (46)	2010	Cross-sectional	West Azerbaijan	Western Iran	Rural	Both	2,503
Basirat et al. (47)	2012	Cross-sectional	Chaharmahal and Bakhtiari	Central Iran	Urban	Both	314
Rafat et al. (48)	2020	Cross-sectional	Tehran	Central Iran	both	Both	720
Dastgiri et al. (49)	2005	Cross-sectional	East Azarbaijan	Western Iran	Urban	Both	300
Esfandiari et al. (50)	2018	Cross-sectional	Tehran	Central Iran	Urban	Both	803
Mahmoudi et al. (51)	2020	Cross-sectional	Kermanshah	Western Iran	Rural	Both	432
Khodabakhshzadeh et al. (52)	2018	Cross-sectional	Kerman	Southern Iran	Rural	Both	384
Tezerji et al. (53)	2020	Cross-sectional	Kerman	Southern Iran	Rural	Both	500
Hosseinpour et al. (54)	2019	Cross-sectional	Tehran	Central Iran	Rural	Both	300
Hakim et al. (55)	2012	Cross-sectional	Khuzestan	Southern Iran	Urban	Both	400
Ziaei et al. (56)	2019	Cross-sectional	Golestan	Northern Iran	Rural	Both	267
Tabibian et al. (57)	2018	Cross-sectional	Tehran	Central Iran	Urban	Female	227
Hamedi-Shahraki et al. (58)	2021	Cross-sectional	Sistan and Baluchestan	Eastern Iran	Urban	Female	630
Shahraki et al. (59)	2016	Cross-sectional	Sistan and Baluchestan	Eastern Iran	both	Both	610
Ramesh et al. (60)	2010	Cross-sectional	Fars	Southern Iran	Urban	Both	778
Gholami et al. (61)	2015	Cross-sectional	Khorasan razavi	Eastern Iran	Rural	Both	4,647
Gholami et al. (62)	2020	Cross-sectional	Tehran	Central Iran	Urban	Both	30,809
Taheri et al. (63)	2016	Cross-sectional	Isfahan	Central Iran	Rural	Both	92
Fallah et al. (64)	2015	Cross-sectional	Yazd	Central Iran	both	Both	500
Kian et al. (65)	2016	Cross-sectional	Alborz	Central Iran	Urban	Both	185
Kian et al. (66)	2020	Cross-sectional	Alborz	Central Iran	Urban	Both	354
Esfarjani et al. (67)	2019	Cross-sectional	Tehran	Central Iran	Urban	Both	630
Jafari et al. (68)	2017	Cross-sectional	Isfahan	Central Iran	Urban	Both	587
Khorramrouz et al. (69)	2020	Cross-sectional	Khorasan razavi	Eastern Iran	Urban	Both	315
Mohammadi et al. (70)	2013	Cross-sectional	Tehran	Central Iran	Urban	Both	418
Pourebrahim et al. (71)	2020	Cross-sectional	Tehran	Central Iran	Urban	Both	583
Rostami et al. (72)	2014	Cross-sectional	Kermanshah	Western Iran	Rural	Both	100
Fallah Tafti et al. (73)	2015	Cross-sectional	Markazi	Central Iran	Urban	Both	300
Safa et al. (74)	2021	Cross-sectional	Zanjan	Western Iran	Rural	Both	353
Nikniaz et al. (75)	2017	Cross-sectional	East Azarbaijan	Western Iran	both	Both	1,277
Nikniaz et al. (76)	2018	Cross-sectional	East Azarbaijan	Western Iran	both	Both	253
Qomi et al. (77)	2012	Cross-sectional	Tehran	Central Iran	Urban	Both	200
Najafianzade et al. (78)	2015	Cross-sectional	Markazi	Central Iran	Rural	Both	373
Safarpour et al. (79)	2014	Cross-sectional	Gilan	Northern Iran	Urban	Female	400
Amiresmaeili et al. (5)	2021	Cross-sectional	Kerman	Southern Iran	Urban	Both	559
EsmaIilnezhad et al. (80)	2018	Cross-sectional	South Khorasan	Eastern Iran	Rural	Both	290
Keshavarz et al. (81)	2021	Cross-sectional	Fars	Southern Iran	Rural	Both	219
Akbarpour et al. (82)	2016	Cross-sectional	Fars	Southern Iran	both	Both	500
Mohammadi et al. (83)	2016	Cross-sectional	Tehran	Central Iran	Urban	Both	33,793
Shakiba et al. (84)	2021	Cross-sectional	Gilan	Northern Iran	Rural	Both	573
Farhangi et al. (85)	2015	Cross-sectional	East Azarbaijan	Western Iran	Urban	Both	300
Sheikhi et al. (86)	2021	Cross-sectional	Sistan and Baluchestan	Eastern Iran	Rural	Both	321
Ebadi-Vanestanagh et al. (87)	2019	Cross-sectional	East Azarbaijan	Western Iran	Urban	Female	188
Savari et al. (88)	2014	Cross-sectional	Kurdistan	Western Iran	Rural	Female	213
Azami et al. (89)	2018	Cross-sectional	Khuzestan	Southern Iran	Rural	Both	101
Payab et al. (90)	2012	Cross-sectional	Tehran	Central Iran	Urban	Female	430
Bagheri et al. (91)	2020	Cross-sectional	Gilan	Northern Iran	Rural	Both	200
Cheraghi et al. (92)	2017	Cross-sectional	Zanjan	Western Iran	Rural	Female	247
Cheraghi et al. (93)	2018	Cross-sectional	Zanjan	Western Iran	Rural	Both	290
Cheraghi et al. (94)	2018	Cross-sectional	Khorasan razavi	Eastern Iran	Rural	Both	304
Sotoudeh et al. (95)	2021	Cross-sectional	Sistan and Baluchestan	Eastern Iran	Urban	Both	421
Eghrari et al. (96)	2020	Cross-sectional	Tehran	Central Iran	Urban	Both	384
Minaie et al. (97)	2019	Cross-sectional			Urban	Both	7,028
Asadi-Lari et al. (98)	2019	Cross-sectional	Tehran	Central Iran	Urban	Both	30,809
Salarkia et al. (99)	2016	Cross-sectional	Tehran	Central Iran	both	Female	423
Omidvar et al. (100)	2019	Cross-sectional			both	Both	1,000
Abedi et al. (101)	2013	Cross-sectional	Khuzestan	Southern Iran	Urban	Both	1,256
Safarpour et al. (102)	2018	Cross-sectional	Gilan	Northern Iran	Urban	Female	400
Arzhang et al. (103)	2019	Cross-sectional	Kermanshah	Western Iran	both	Both	364
Ekhlaspour et al. (104)	2019	Cross-sectional	Kerman	Southern Iran	Urban	Both	700
Alipour et al. (105)	2021	Cross-sectional	Tehran	Central Iran	Urban	Both	1,000
Mortazavi et al. (106)	2017	Cross-sectional	Sistan and Baluchestan	Eastern Iran	Urban	Both	2,160
Dassie et al. (107)	2016	Cross-sectional	Tehran	Central Iran	Urban	Both	644
Abdar-Esfehani et al. (108)	2019	Master tehsis	Tehran	Central Iran	Urban	Both	586
Razzazi et al. (109)	2014	Master tehsis	Qazvin	Central Iran	Urban	Female	250
Kazemi et al. (110)	2018	Master tehsis	Ardabil	Western Iran	Urban	Male	324
Darini et al. (111)	2016	Master tehsis	Kerman	Southern Iran	both	Both	400
Sharaki et al. (112)	2015	Cross-sectional	Chaharmahal and Bakhtiari	Central Iran	Rural	Both	273
Siasar et al. (113)	2017	Master tehsis	Sistan and Baluchestan	Eastern Iran	both	Both	301
Nadimi et al. (114)	2017	Master thesis	Kurdistan	Western Iran	Rural	Both	296
Rahimi-Moghaddam et al. (115)	2015	Cross-sectional	Lorestan	Western Iran	both	Both	200
Abbasi et al. (116)	2019	Cross-sectional	Gilan	Northern Iran	Rural	Both	98
Esa pare et al. (117)	2016	Master thesis	Khuzestan	Southern Iran	Rural	Both	460
Forootan et al. (118)	2013	Master thesis	Kermanshah	Western Iran	Rural	Female	60
Pakravan-Charvadeh et al. (119)	2021	Cross-sectional	Khuzestan	Southern Iran	both	Both	200
Rezvani et al. (120)	2018	Cross-sectional	Kohgiluyeh and Boyer-Ahmad	Southern Iran	Rural	Both	325
Hashemitabar et al. (121)	2018	Cross-sectional	Kerman	Southern Iran	Rural	Both	400
Bayanani et al. (122)	2020	Master thesis	Hamedan	Western Iran	Rural	Both	388
Parsay et al. (123)	2018	Master thesis	East Azarbaijan	Western Iran	Urban	Both	400
Moradi et al. (124)	2020	Cross-sectional	Kermanshah	Western Iran	Urban	Both	217
Amin et al. (125)	2021	Cross-sectional	Isfahan	Central Iran	Urban	Both	358
Jamini et al. (126)	2016	Cross-sectional	Kermanshah	Western Iran	Rural	Both	180
Ahmadi Dehrashid et al. (127)	2021	Cross-sectional	Kurdistan	Western Iran	Rural	Both	60
Abbasi et al. (128)	2016	Cross-sectional	Alborz	Central Iran	Rural	Both	166

**Figure 2:**
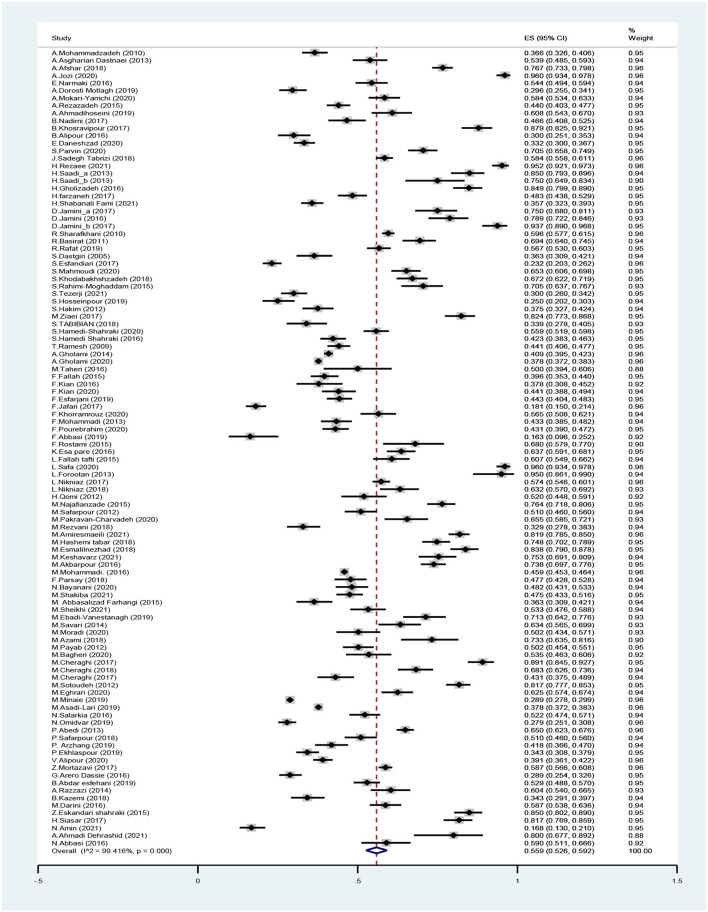
Forest plot for the prevalence of food insecurity in Iran.

According to the meta-analysis, the total prevalence of FI in Iran was 55.9% (95% CI: 52.6–59.2%) (Figure 2). To determine heterogeneity, meta-regression and subgroup analyses were conducted.

### Subgroups and meta-regression analyses

The prevalence of FI in rural areas was 66.1% (95% CI: 58.8–73.4%) which was higher than the prevalence of FI in urban areas [47.1% (95% CI: 44.1–50.0%)] (Supplementary Figure 1). The prevalence of FI among women was higher [51.3% (95% CI: 45.1–57.6%) than the prevalence among men (47.8% (95% CI: 41.8–53.8%)] (Supplementary Figure 2). Pooled prevalence of FI was 44.7% (95% CI: 35.5–53.9%) in children, 40.6% (95% CI: 33.2–48.0%) in adolescents, 56.5% (95% CI: 51.7–61.2%) in adults, and 49.8 % (95% CI: 36.6–63.0%) in older adults.

**Figure 3:**
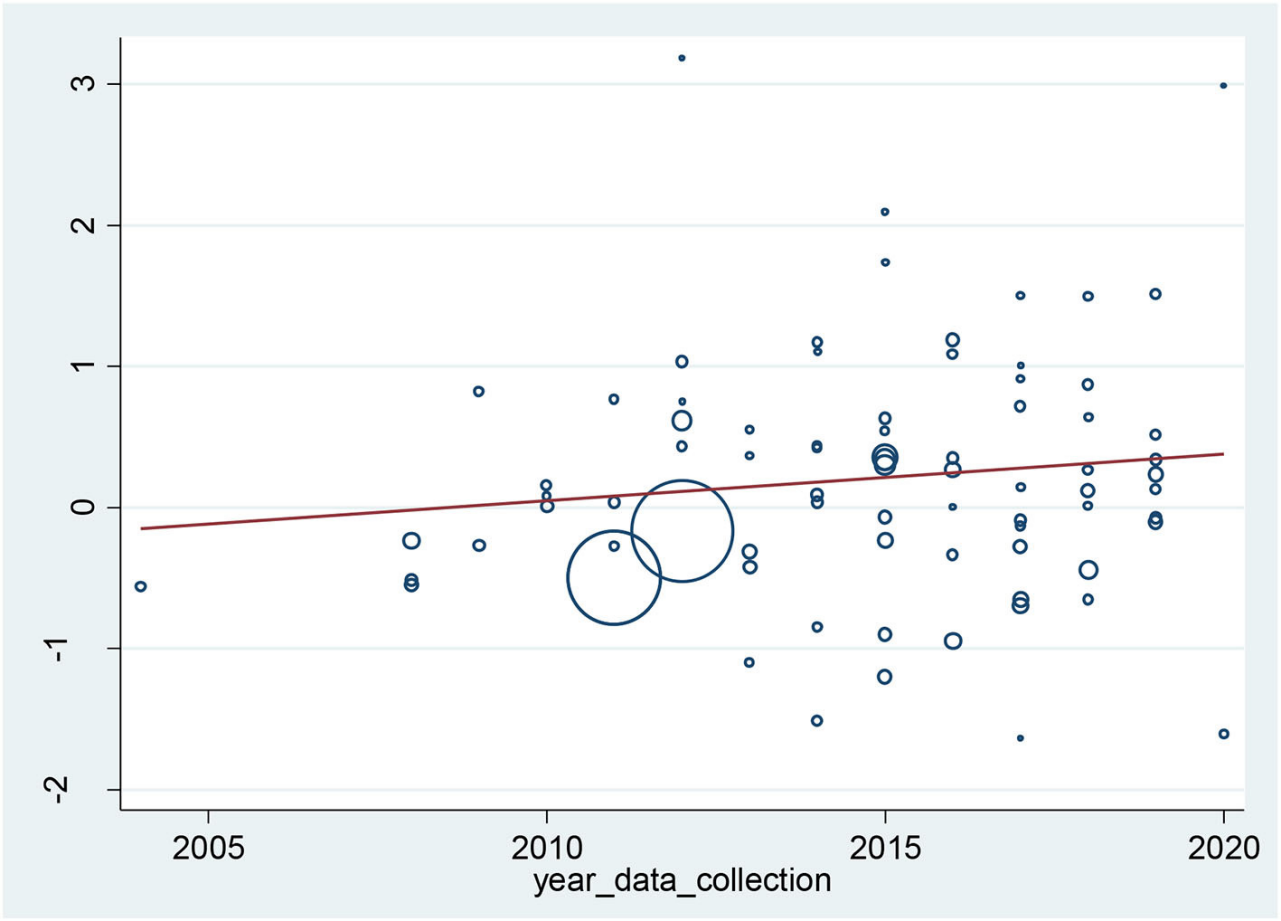
Association of the prevalence of food insecurity in households with years of data collection.

Subgroup analyses by region revealed that the highest prevalence of FI was in the western part of the country [64.8% (95% CI: 57.7–72.0%)] and the lowest prevalence of FI was in the central part of the country [46.1% (95% CI: 43.1–49.2%)]. The prevalence of FI was also assessed according to questionnaires used in the studies. The prevalence of FI in studies that used USDA 18-item was 54.8% (95% CI: 49.0–60.6%), the HFIAS was 58.4% (95% CI: 49.8–67.1%), and the USDA 6-item was 48.2% (95% CI: 44.3–52.0 %). The highest prevalence of FI was 77.8 % (95% CI: 69.1–93.8%) in studies that used the Coping Strategies Index. Moreover, the prevalence was 53.4% (95% CI: 43.0–63.7%) in studies that used FaCPS-FSSM and 63.2% (95% CI: 38.2–88.1%) in studies that used Radimer-Cornell (Table 2). Meta-regression analyses using random effects models were conducted to analyze the change in the prevalence of FI over time; however, results indicated that there were no significant changes in FI over time (Figure 3).

**Table 2:** Food insecurity prevalence data by location, sex, region, age group, quality of studies, and assessment tool.

**Group**	**Subgroups**	**Number of studies**	**Sample size**	**Prevalence (%)**	**95% CI**	**Heterogeneity**		**Heterogeneity between subgroups**
						***I*** **^2^**	***Q***	***P*** **-value**
Location	Rural	41	17,771	66.1	58.8–73.4	99.28	< 0.000	< 0.000
	Urban	51	127,643	47.1	44.1–50.0	98.99	< 0.000	
	both	14	6,946	58.5	48.9–68.1	98.65	< 0.000	
Gender	Male	20	9,499	47.8	41.8–53.8	96.93	< 0.000	0.014
	Female	35	13,835	51.3	45.1–57.6	98.32	< 0.000	
	both	70	129,896	58.1	54.0–62.3	99.54	< 0.000	
Region	Central	36	110,498	46.1	43.1–49.2	98.80	< 0.000	< 0.000
	East	11	10,239	59.9	49.9–69.8	98.96	< 0.000	
	North	7	2,229	56.8	36.2–77.4	99.33	< 0.000	
	South	16	7,582	59.2	50.4–68.0	98.58	< 0.000	
	West	34	13,724	64.8	57.7–72.0	99.04	< 0.000	
Type of questionnaire	USDA 18-item	48	19,608	54.8	49.0–60.6	98.77	< 0.000	0.003
	HFIAS	32	19,110	58.4	49.8–67.1	99.26	< 0.000	
	USDA 6-item	12	107,461	48.2	44.3–52.0	99.23	< 0.000	
	Radimer-Cornell	6	2,160	63.2	38.2–88.1	99.50	< 0.000	
	FaCPS-FSSM	3	1,603	53.4	43.0–63.7	.	.	
	Coping Strategies Index	4	1,358	77.8	69.1–93.8	98.52	< 0.000	
Age group	Children	14	13,011	44.7	35.5–53.9	99.01	< 0.000	0.002
	Adolescents	6	2,786	40.6	33.2–48.0	93.98	< 0.000	
	Adults	45	81,026	56.5	51.7–61.2	99.34	< 0.000	
	Older adults	9	4,482	49.8	36.6–63.0	99.21	< 0.000	
Quality of studies	High quality	70	63,584	54.9	49.9–59.9	99.34	< 0.000	0.386
	Low quality	36	88,716	57.8	52.6–59.2	99.33		
All studies		106	152,300	55.9	52.6–59.2	99.42	< 0.000	-

### Quality assessment

The Joanna Briggs Institute (JBI) quality assessment checklist was used to assess the quality of the published articles (19). According to the checklist, 70 studies were categorized as high quality and 36 studies were categorized as low quality (Supplementary Table 2). Subgroup analysis revealed no evidence of a statistical difference between the two groups.

### Publication bias

**Figure 4:**
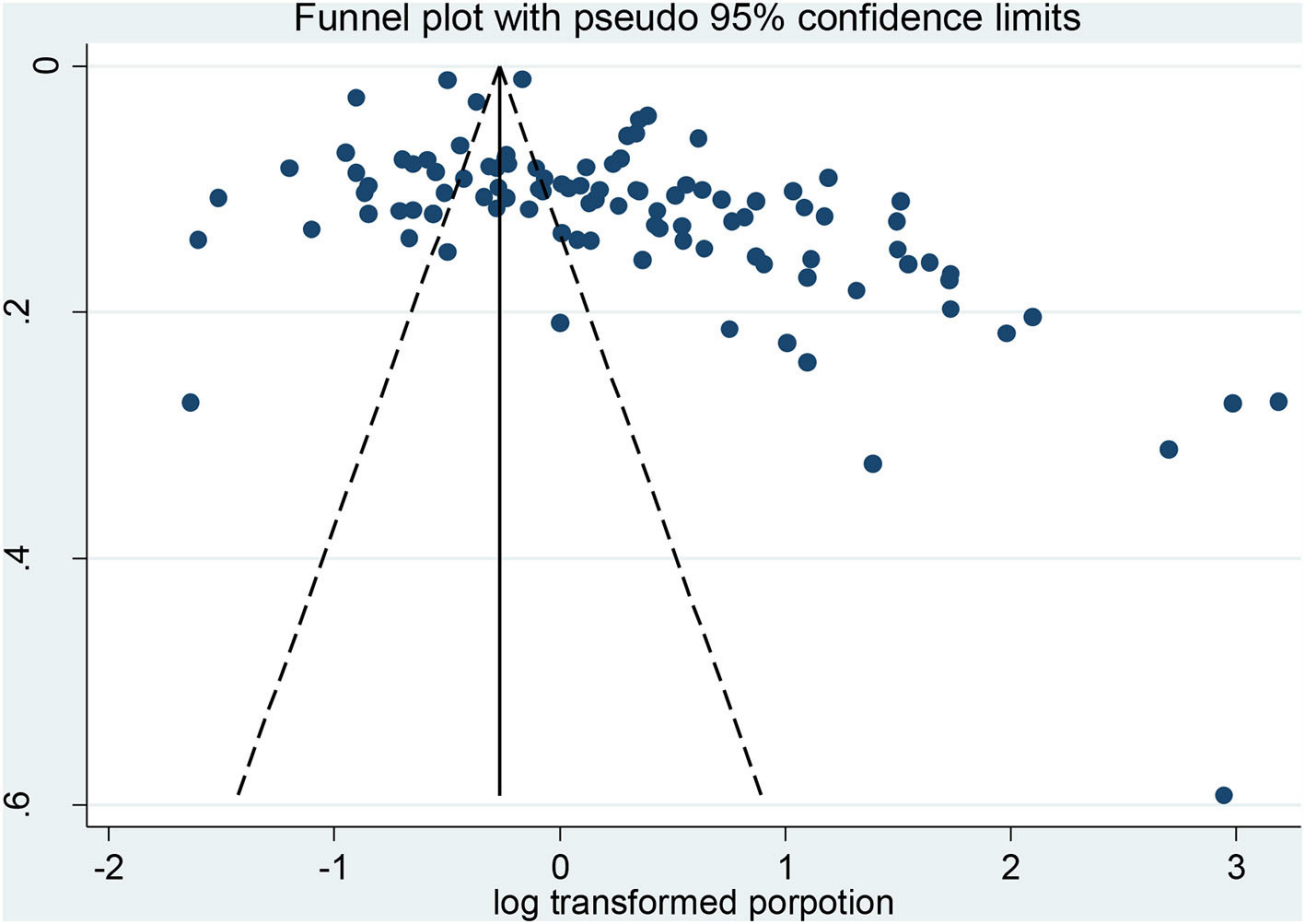
Funnel plot of included studies.

**Figure 5:**
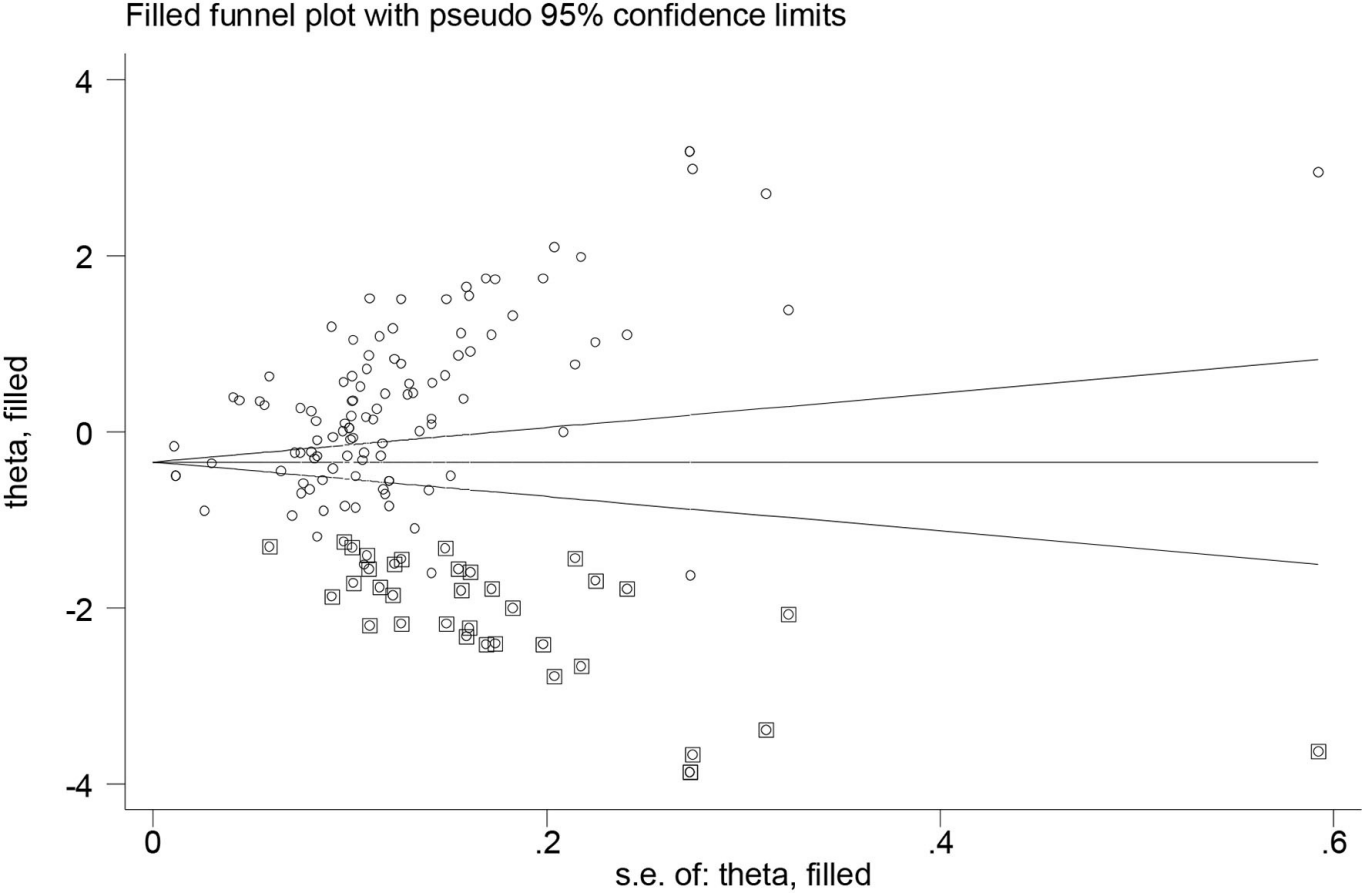
Funnel plot for publication bias with trim and fill method.

Despite searching several databases, searching gray literature, and not having language limitations, the results of the funnel plot (Figure 4) and Egger's test (*P* < 0.000) revealed evidence of publication bias. The trim and fill method using random effects was used to adjust the pooled prevalence estimates for publication bias. After imputing 36 studies below the mean, the result indicated an adjusted prevalence of 74.6% (95% CI: 67.3–82.7%) among participants (Figure 5). Furthermore, there was a significant heterogeneity between subgroups based on the region (*P-*value < 0.000), gender (*P-*value = 0.017), type of FI questionnaire (*P-*value = 0.004), and location (*P-*value < 0.000).

### Sensitivity analysis

Sensitivity analysis suggested that there were no changes in the mean prevalence after the removal of individual studies (Supplementary Figure 3).

## Discussion

The present systematic review and meta-analysis evaluated the prevalence of FI in Iran examining 106 articles and theses among 152,300 participants from 2010 to 2021 with the consideration of gender, geographical areas, age groups, location, and type of questionnaires. According to the United Nations International Children's Emergency Fund (UNICEF), about 375.8 million people in Asian countries including Iran and the Pacific faced hunger in 2020, which increased by nearly 54 million people compared to 2019. Moreover, more than 1.1 billion people did not have access to adequate food in 2020 (129). Based on the present meta-analysis, the total prevalence of FI in Iran was 55.9% (95% CI: 52.6–59.2%), which is considered high. A previous meta-analysis evaluating FI in Iran also revealed a high prevalence of FI among different age categories (6, 11, 12). Differences in inclusion and exclusion criteria could be considered a potential reason for the contrasting findings. Additionally, this present study evaluated a greater amount of studies over several databases using a comprehensive search strategy.

In this study, subgroup analyses were performed based on identified factors to evaluate FI prevalence comprehensively. Subgroup analyses revealed that FI prevalence was significantly higher in Iranian women compared to men (51.3 vs. 47.8%, *p* < 0.000). In line with our findings, Diab-El-Harake et al. found a higher prevalence of FI in women living in Arab countries (130). In addition, a meta-analysis conducted by Jung et al. found that female-headed households were 75% (95 % CI: 49–96%) more likely to experience FI compared to male-headed households (131). Another meta-analysis in Ethiopia also found a higher FI prevalence in female-headed households (132). Furthermore, based on the 2019 State of FI report, the prevalence of FI was higher among women compared to men in every continent from 2016 to 2019 (133). Broussard (134) suggested that a higher prevalence of FI may be explained by differences in household income, social networks, and educational attainment. Furthermore, that gender differences in income accounted for more than 70% of the gender gap in FI, and lower educational attainment accounted for 5–45% of the gap. In addition, women may express more positive emotions when they are not necessarily experiencing favorable conditions, suggesting that cultural factors may influence the status of FI in women (135).

According to Sinclair et al., rural women had a higher prevalence of FI, which is in accordance with our subgroup analyses based on the region that indicated that the prevalence of FI was higher in rural areas [66.1% (95% CI: 58.8–73.4%)] compared to urban areas [47.1% (95% CI: 58.8–73.4%)] (136). Another study conducted by Sims et al. found that there was FI and malnutrition among Indian rural women (137). In addition, Sansón-Rosas et al. found high rates of FI in Colombian rural households (138). It is accepted that poverty is significantly linked to FI (139) and it is often considered the most common determinant of FI across the globe (139). Economic constraints may explain a higher prevalence of FI in rural areas (140). Many people who are from low-income households and are vulnerable to FI reside in rural areas and depend on agricultural activities to produce food; and in case of limited access to agricultural facilities and lands, they will face inadequate food availability (141).

In the present study, the highest prevalence of FI was among adults (56.5% [95% CI: 51.7–61.2%]) compared to different age groups. Sinclair et al. also found a higher prevalence of FI among people between the ages of 25–49 across the globe (136). A possible explanation for these findings may be that adults are often the head of the household and in the case of inadequate access to food, they may prioritize other members of the household. It also should be considered that fewer studies have been conducted among adolescents and older adults compared to those conducted on the adult population in Iran.

Another factor in the present study that made a significant difference in the prevalence of FI was the location of the participants' habitation as it was found that FI was higher in the western part of the country (64.8% [95% CI: 57.7–72.0%]) compared to the eastern (59.9% [95% CI: 49.9–69.8%]) and southern (59.2% [95% CI: 50.4–68.0%]) parts of the country. Mortazavi et al. conducted a cross-sectional study evaluating FI in the southern parts of Iran and in accordance with results from the present study, found a high prevalence of FI (58.8 %) (142). The current study found that the prevalence of FI in the northern parts of Iran was 56.8% (95% CI: 36.2–77.4%). A cross-sectional study conducted by Shakiba et al. also found a high prevalence of FI (50.8%) in northern Iran (84). Studies have shown that there are many risk factors for FI, including demographic characteristics and financial resources including age, economic status, employment, savings, educational level of the head of the household, single parenthood, and ethnicity (84, 142, 143). The impact of these risk factors on food security varies across populations, which reveals the potential influence of regional practices, policies, and nutrition programs (144, 145). However, to our knowledge, no study to date has examined the impact of regional influences in Iran. Future research is needed to validate the types of conclusions that can be drawn from this study.

Subgroup analyses were also conducted based on the type of questionnaire used to evaluate FI. Despite the fact that the questionnaires used in all of the studies were validated, there was evidence of a statistically significant difference between subgroups (*p-*value for heterogeneity: 0.003). However, the results for the prevalence of FI from the USDA 18-item (54.8% [95% CI: 49.0–60.6%]) and HFIAS (58.4% [95% CI: 49.8–67.1%]) were relatively similar, which were the questionnaires used in most of the studies. Other questionnaires were only used in a small number of included studies, which may explain the significant differences that were found in this study in the subgroup analysis for the type of questionnaire. Finally, the subgroup analysis for the results of the quality assessment did not reveal any evidence of a significant difference between high-quality and low-quality studies.

### Strengths and limitation

To the best of our knowledge, the current meta-analysis included the highest number of studies to evaluate the prevalence of FI in Iran. The previous meta-analysis conducted by Behzadifar et al. (129) included 31 studies until 2015; therefore, the prevalence estimates reported in the present study represent the current findings. In addition, we performed subgroup analyses based on previously identified factors that may influence FI and found significant associations between the named factors and the prevalence of FI, which provided a strong comprehensive review of the relationships. However, this study had limitations worth considering when evaluating the findings. There was evidence of high heterogeneity between included studies. Furthermore, we did not assess the different levels of FI that may exist among the participants. Future studies and reviews may consider evaluating varying levels of FI as it may provide more information about FI status.

## Conclusion

The findings of this systematic review and meta-analysis revealed that the prevalence of FI is high in Iran. The prevalence was higher among women, adults (aged), in rural areas and the western part of the country. It is an important finding that the prevalence of FI in Iran is influenced by age, gender, socioeconomic status, and habitation.

## Data availability statement

The original contributions presented in the study are included in the article/Supplementary material, further inquiries can be directed to the corresponding author.

## Author contributions

LA and PA contributed to the study design, registered the study protocol, manuscript revision, and final version approval. NB edited the manuscript. SA, PS, MM, and MN-S contributed to the literature searches, title/abstract and full-text screening, extracted data, quality assessment, and manuscript drafting. PA contributed to the statistical analysis, ran the meta-analyses, and did data charting. All authors contributed to the article and approved the submitted version.

## Funding

This study was supported by the Tehran University of Medical Sciences (Grant Number: IR.TUMS.MEDICINE.REC.1400.1423).

## Conflict of interest

The authors declare that the research was conducted in the absence of any commercial or financial relationships that could be construed as a potential conflict of interest.

## Publisher's note

All claims expressed in this article are solely those of the authors and do not necessarily represent those of their affiliated organizations, or those of the publisher, the editors and the reviewers. Any product that may be evaluated in this article, or claim that may be made by its manufacturer, is not guaranteed or endorsed by the publisher.
