# Supplementary material for: Prevalence of household food insecurity among a healthy Iranian population: A systematic review and meta-analysis
Source: Front Nutr. 2022 Nov 15;9:1006543. doi: 10.3389/fnut.2022.1006543 (PMC9707736; doi:10.3389/fnut.2022.1006543)
Supplement: Supplementary file 1 [file Data_Sheet_1.docx]

**Supplementary material:**

**Supplementary Table 1.** Detailed search terms in the databases

| **Database** | **Search term** |
| --- | --- |
| Pubmed | (food[Title/Abstract] AND Supply[Title/Abstract]) OR (food[Title/Abstract] AND Supplies[Title/Abstract]) OR (food[Title/Abstract] AND insecurit*[Title/Abstract]) OR (food[Title/Abstract] AND securit*[Title/Abstract]) OR (food[Title/Abstract] AND insufficiency[Title/Abstract]) AND (iran*[tiab] OR iran[pl] OR iran[ad] OR Persia*[tiab])  AND (1990/01/01:2022/03/01[dp]) |
| Scopus | (( TITLE-ABS-KEY ( food AND supply ) OR TITLE-ABS-KEY ( food AND supplies ) OR  TITLE-ABS-KEY ( food AND insecurit* ) OR TITLE-ABS-KEY ( food AND securit* ) OR  TITLE-ABS-KEY ( food AND insufficiency )) AND (TITLE-ABS-KEY (iran*) OR  AFFILCOUNTRY(iran) OR AFFIL(iran) OR PUBLISHER(iran) OR  TITLE-ABS-KEY (Persia*))) AND ( ( PUBYEAR > 1990 AND PUBYEAR < 2022 ) OR PUBDATETXT ( "January 2021" OR "February 2021") ) |
| ISI Web of Science | ((TS=( food AND supply) OR TS=( food AND supplies) OR TS=( food AND insecurit*) OR TS=( food AND securit*) OR TS=( food AND insufficiency) ) AND (TS=( iran*) OR  AD=(iran) OR TI=(iran) OR FT=(iran) OR  TS=( Persia*))) AND (PY=(1990-2022)) |
| Iranian databases | Food insecurity AND Food security |

| Supplementary Table 2: Results of quality assessment of studies using the Joanna Briggs Institute (JBI) Critical Appraisal Checklist for Cross Sectional Studies. | | | | | | | | | | |
| --- | --- | --- | --- | --- | --- | --- | --- | --- | --- | --- |
| authors | JBI_Q1 | JBI_Q2 | JBI_Q3 | JBI_Q4 | JBI_Q5 | JBI_Q6 | JBI_Q7 | JBI_Q8 | JBI_Q9 | JBI Total Score |
| A.Mohammadzadeh | Yes | Yes | Unclear | Yes | Unclear | Yes | Yes | Yes | Unclear | low risk |
| A.Asgharian Dastnaei | Unclear | Yes | Yes | Yes | Unclear | Yes | Yes | Yes | Unclear | low risk |
| A.Afshar | Yes | Yes | Yes | Yes | Unclear | Yes | Yes | Yes | Unclear | low risk |
| A.Jozi | Unclear | Yes | Yes | Yes | Yes | Yes | Yes | Yes | Yes | low risk |
| E.Narmaki | Unclear | Yes | Unclear | Yes | Unclear | Yes | Yes | Yes | Unclear | low risk |
| A.Dorosti Motlagh | Unclear | Yes | Unclear | Yes | Unclear | Yes | Unclear | Yes | Unclear | high risk |
| A.Mokari-Yamchi | Unclear | Yes | Unclear | Yes | Unclear | Yes | Unclear | Yes | Unclear | high risk |
| A.Rezazadeh | Yes | Yes | Unclear | Yes | Unclear | Yes | Unclear | Yes | Unclear | low risk |
| A.Ahmadihoseini | Yes | Yes | Unclear | Yes | Unclear | Yes | Unclear | Yes | Unclear | low risk |
| B.Nadimi | Yes | Yes | Yes | Yes | Unclear | Yes | Yes | Yes | Unclear | low risk |
| B.Khosravipour | Unclear | No | Yes | Yes | Unclear | Yes | Yes | Yes | Unclear | low risk |
| B.Alipour | Yes | Yes | Yes | Yes | Unclear | Yes | Unclear | Yes | Unclear | low risk |
| E.Daneshzad | Yes | Yes | Unclear | Yes | Unclear | Yes | Unclear | Yes | Unclear | low risk |
| S.Parvin | Yes | Yes | Unclear | Yes | Unclear | Yes | Yes | Yes | Unclear | low risk |
| J.Sadegh Tabrizi | Yes | Yes | Yes | Yes | Yes | Yes | Unclear | Yes | Yes | low risk |
| H.Rezaee | Unclear | Yes | Yes | Yes | Yes | Unclear | Unclear | Yes | Yes | low risk |
| H.Saadi_a | Unclear | Yes | Yes | Yes | Unclear | Yes | Yes | Yes | Unclear | low risk |
| H.Saadi_b | Unclear | Yes | Yes | Yes | Unclear | Yes | Yes | Yes | Unclear | low risk |
| H.Gholizadeh | Unclear | Yes | Yes | Yes | Unclear | Yes | Yes | Yes | Unclear | low risk |
| H.farzaneh | Yes | Yes | Yes | Yes | Unclear | Yes | Unclear | Yes | Unclear | low risk |
| H.Shabanali Fami | Yes | Yes | Yes | Yes | Unclear | Yes | Yes | Yes | Unclear | low risk |
| D.Jamini_a | Unclear | Yes | Yes | Yes | Unclear | Yes | Yes | Yes | Unclear | low risk |
| D.Jamini | Unclear | Yes | Yes | Yes | Yes | Yes | Yes | Yes | Yes | low risk |
| D.Jamini_b | Unclear | Yes | Yes | Yes | Unclear | Yes | Yes | Yes | Unclear | low risk |
| R.Sharafkhani | Unclear | Unclear | Unclear | Yes | Unclear | Yes | Unclear | Yes | Unclear | high risk |
| R.Basirat | Yes | Yes | Yes | Yes | Yes | Yes | Yes | Yes | Yes | low risk |
| R.Rafat | Yes | Yes | Unclear | Yes | Unclear | Yes | Unclear | Yes | Unclear | low risk |
| S.Dastgiri | Yes | No | Unclear | Yes | Unclear | Yes | Yes | Yes | Unclear | low risk |
| S.Esfandiari | Yes | Yes | Unclear | Yes | Unclear | Yes | Unclear | Yes | Unclear | low risk |
| S.Mahmoudi | Unclear | Yes | Unclear | Yes | Unclear | Unclear | Unclear | Yes | Unclear | high risk |
| S.Khodabakhshzadeh | Unclear | Yes | Yes | Yes | Unclear | Yes | Yes | Yes | Unclear | low risk |
| S.Rahimi-Moghaddam | Unclear | Yes | Yes | Yes | Unclear | Yes | Unclear | Yes | Unclear | low risk |
| S.Tezerji | Yes | Yes | Yes | Yes | Unclear | Yes | Yes | Yes | Unclear | low risk |
| S.Hosseinpour | Yes | Yes | Yes | Yes | Unclear | Yes | Unclear | Yes | Unclear | low risk |
| S.Hakim | Yes | Yes | Yes | Yes | Unclear | Yes | Yes | Yes | Unclear | low risk |
| M.Ziaei | Unclear | Yes | Yes | Yes | Unclear | Yes | Yes | Yes | Unclear | low risk |
| S.TABIBIAN | Yes | Yes | Unclear | Yes | Unclear | Yes | Unclear | Yes | Unclear | low risk |
| S.Hamedi-Shahraki | Yes | Yes | Yes | Yes | Unclear | Yes | Yes | Yes | Unclear | low risk |
| S.Hamedi Shahraki | Yes | Yes | Yes | Yes | Unclear | Yes | Unclear | Yes | Unclear | low risk |
| T.Ramesh | Yes | Yes | Unclear | Yes | Unclear | Yes | Unclear | Yes | Unclear | low risk |
| A.Gholami | Unclear | Yes | Unclear | Yes | Unclear | Yes | Unclear | Yes | Unclear | high risk |
| A.Gholami | Unclear | Yes | Unclear | Yes | Unclear | Yes | Unclear | Yes | Unclear | high risk |
| M.Taheri | Yes | Yes | Yes | Yes | Yes | Unclear | Unclear | Yes | Yes | low risk |
| F.Fallah | Yes | Yes | Yes | Yes | Unclear | Yes | Yes | Yes | Unclear | low risk |
| F.Kian | Unclear | Yes | Yes | Yes | No | Yes | Yes | Yes | No | low risk |
| F.Kian | Unclear | Yes | Yes | Yes | Unclear | Yes | Yes | Yes | Unclear | low risk |
| F.Esfarjani | Unclear | Yes | Unclear | Yes | Yes | Yes | Unclear | Yes | Yes | low risk |
| F.Jafari | No | Yes | Yes | Yes | No | Yes | Yes | No | No | low risk |
| F.Khorramrouz | Unclear | Yes | Unclear | Yes | Unclear | Yes | Unclear | Yes | Unclear | high risk |
| F.Mohammadi | Unclear | Yes | Yes | Yes | Yes | Yes | Yes | Yes | Yes | low risk |
| F.Pourebrahim | Unclear | Yes | Yes | Yes | Yes | Yes | Unclear | Yes | Yes | low risk |
| F.Abbasi | Unclear | Yes | Yes | Yes | Yes | Unclear | Unclear | Yes | Yes | low risk |
| F.Rostami | Unclear | Yes | Unclear | Yes | Yes | Yes | Unclear | Yes | Yes | low risk |
| K.Esa pare | Unclear | Yes | Unclear | Yes | No | Yes | Yes | Yes | Yes | low risk |
| L.Fallah tafti | Unclear | Yes | Unclear | Yes | Unclear | Unclear | Unclear | Yes | Unclear | high risk |
| L.Safa | Unclear | Yes | Unclear | Yes | No | Yes | Yes | Yes | No | low risk |
| L.Forootan | Unclear | No | Unclear | Yes | Unclear | Yes | Yes | Yes | Unclear | high risk |
| L.Nikniaz | Unclear | Yes | Yes | Yes | No | Yes | Unclear | Yes | No | low risk |
| L.Nikniaz | Unclear | Yes | Yes | Yes | No | Yes | Unclear | Yes | No | low risk |
| H.Qomi | Unclear | Yes | Unclear | Yes | Unclear | Unclear | Unclear | Yes | Unclear | high risk |
| M.Najafianzade | Unclear | Yes | Yes | Yes | Unclear | Unclear | Unclear | Yes | Unclear | high risk |
| M.Safarpour | Unclear | Unclear | Unclear | Yes | Unclear | Unclear | Unclear | Yes | Unclear | high risk |
| M.PakravanCharvadeh | Unclear | Unclear | Unclear | Yes | Unclear | Unclear | Unclear | Yes | Unclear | high risk |
| M.Rezvani | Unclear | Unclear | Unclear | Yes | Unclear | Unclear | Yes | Yes | Unclear | high risk |
| M.Amiresmaeili | Yes | Yes | Yes | Yes | No | Yes | Unclear | Yes | No | low risk |
| M.Hashemi tabar | Unclear | Yes | Yes | Yes | Unclear | Unclear | Unclear | Yes | Unclear | high risk |
| M.EsmaIilnezhad | Unclear | Yes | Yes | Yes | Unclear | Unclear | Unclear | Yes | Unclear | high risk |
| M.Keshavarz | Unclear | Yes | Yes | No | Unclear | Yes | Yes | Yes | Unclear | low risk |
| M.Akbarpour | Unclear | Yes | Yes | Yes | Unclear | Unclear | Yes | Yes | Unclear | low risk |
| M.Mohammadi. | Unclear | Yes | Yes | Yes | Yes | Unclear | Unclear | Yes | Yes | low risk |
| F.Parsay | Unclear | Yes | Yes | Yes | Unclear | Yes | Yes | Yes | Unclear | low risk |
| N.Bayanani | Unclear | Unclear | Unclear | Yes | Unclear | Yes | Yes | Yes | Unclear | high risk |
| M.Shakiba | Unclear | Yes | Yes | Yes | Unclear | Yes | Yes | Yes | No | low risk |
| M. Abbasalizad Farhangi | No | No | Unclear | No | Unclear | Unclear | No | Yes | Unclear | high risk |
| M.Sheikhi | No | Unclear | Unclear | Yes | Unclear | Yes | Unclear | Yes | Unclear | high risk |
| M.Ebadi-Vanestanagh | Unclear | Yes | Unclear | No | Unclear | Yes | Unclear | Yes | Unclear | high risk |
| M.Savari | Unclear | Yes | Unclear | Yes | Unclear | Yes | Unclear | Yes | Unclear | low risk |
| M.Moradi | Unclear | Unclear | Yes | No | Unclear | Unclear | Unclear | Yes | Unclear | high risk |
| M.Azami | Unclear | Yes | Unclear | No | Unclear | Yes | Yes | Yes | Unclear | high risk |
| M.Payab | Yes | Yes | Yes | Yes | Unclear | Yes | No | Yes | Unclear | low risk |
| M.Bagheri | Unclear | Yes | Unclear | Yes | Unclear | Yes | Unclear | Yes | Unclear | high risk |
| M.Cheraghi | Unclear | Yes | Unclear | Yes | Unclear | Yes | Unclear | Yes | Unclear | high risk |
| M.Cheraghi | Unclear | Yes | Unclear | Yes | Unclear | Unclear | Unclear | Yes | Unclear | high risk |
| M.Cheraghi | Unclear | Yes | Unclear | Yes | Unclear | Yes | Yes | Yes | Unclear | low risk |
| M.Sotoudeh | Unclear | Yes | Yes | Yes | Unclear | Yes | Unclear | Yes | Unclear | low risk |
| M.Eghrari | Yes | Yes | Unclear | Yes | Unclear | Yes | Yes | Yes | Unclear | low risk |
| M.Minaie | Unclear | Yes | Unclear | No | Unclear | Unclear | Unclear | No | Unclear | high risk |
| M.Asadi-Lari | Unclear | Unclear | Unclear | No | Unclear | Yes | No | No | Unclear | high risk |
| N.Salarkia | Unclear | Unclear | Unclear | Unclear | Unclear | Yes | Unclear | Yes | Unclear | high risk |
| N.Omidvar | Unclear | Unclear | Unclear | No | Unclear | Yes | Unclear | No | Unclear | high risk |
| P.Abedi | Unclear | Unclear | Unclear | No | Unclear | Yes | Yes | Yes | Unclear | high risk |
| P.Safarpour | Unclear | Yes | Unclear | Yes | Unclear | Yes | Yes | Yes | Unclear | low risk |
| P. Arzhang | Yes | Yes | Unclear | Yes | Unclear | Yes | Yes | Yes | Unclear | low risk |
| P.Ekhlaspour | Unclear | Yes | Unclear | Yes | Unclear | Yes | Yes | Yes | Unclear | low risk |
| V.Alipour | Unclear | Yes | Yes | Yes | Unclear | Yes | Unclear | Yes | Unclear | low risk |
| Z.Mortazavi | Unclear | Unclear | Unclear | Unclear | Unclear | Yes | Unclear | Yes | Unclear | high risk |
| G.Arero Dassie | Unclear | Yes | Unclear | Yes | Unclear | Yes | Yes | Unclear | Unclear | high risk |
| B.Abdar esfehani | Yes | Yes | Yes | Yes | Yes | Yes | Yes | Yes | Yes | low risk |
| A.Razzazi | Unclear | Yes | Yes | Yes | Unclear | Yes | Unclear | Yes | Unclear | low risk |
| B.Kazemi | Yes | Yes | Yes | Yes | Unclear | Yes | Yes | Yes | Unclear | low risk |
| M.Darini | Unclear | Yes | Unclear | Yes | Unclear | Yes | Yes | No | Unclear | high risk |
| Z.Eskandari shahraki | No | Yes | Unclear | Yes | Unclear | Yes | Yes | No | Unclear | high risk |
| H.Siasar | Unclear | Yes | Unclear | Yes | Unclear | Yes | Unclear | Yes | Unclear | high risk |
| N.Amin | Unclear | Yes | Yes | Yes | Unclear | Yes | Unclear | Yes | Unclear | low risk |
| A.Ahmadi Dehrashid | No | Yes | Unclear | Yes | Unclear | Yes | Yes | No | Unclear | high risk |
| N.Abbasi | Yes | Yes | Unclear | Yes | Unclear | Yes | Yes | Yes | Unclear | low risk |

**Supplementary Figure 1.** Forest plot for prevalence of food insecurity in Iran by location

Supplementary Figure 2. Forest plot for prevalence of food insecurity in Iran by sex.

**Supplementary Figure 3.** Forest plot showing the prevalence of food insecurity and 95 % CI from sensitivity analyses in which the given named study is omitted. Meta-analysis estimates of the prevalence of food insecurity and 95 % CI with the given named study omitted are represented by the open circle and the dotted horizontal line, respectively; the solid vertical lines (from left to right) represent the lower 95 % CI, prevalence of food insecurity and the upper 95 % CI for all included studies.
